# P-1412. HIV Retention in Care and Viral Suppression among Transgender Women in a Large Ryan White Clinic

**DOI:** 10.1093/ofid/ofae631.1587

**Published:** 2025-01-29

**Authors:** Danielle L Gilbert, Hope Oddo Moise, Lauren Richey

**Affiliations:** Louisiana State University Health Sciences Center, Section of Infectious Diseases, New Orleans, Louisiana; LSU Health New Orleans, New Orleans, Louisiana; LSU Health Sciences Center New Orleans, New Orleans, Louisiana

## Abstract

**Background:**

Transgender women (TW) are disproportionately affected by HIV with 1 in 5 TW in the United States living with HIV. TW with HIV (TWH) have lower rates of retention and viral suppression than cisgender women and cisgender men, resulting in worse health outcomes and increased HIV transmission. The aim of this quality improvement initiative was to identify TWH at a large urban Ryan White clinic in New Orleans to guide future quality improvement efforts to improve retention and viral suppression of TWH.

**Table 1.**
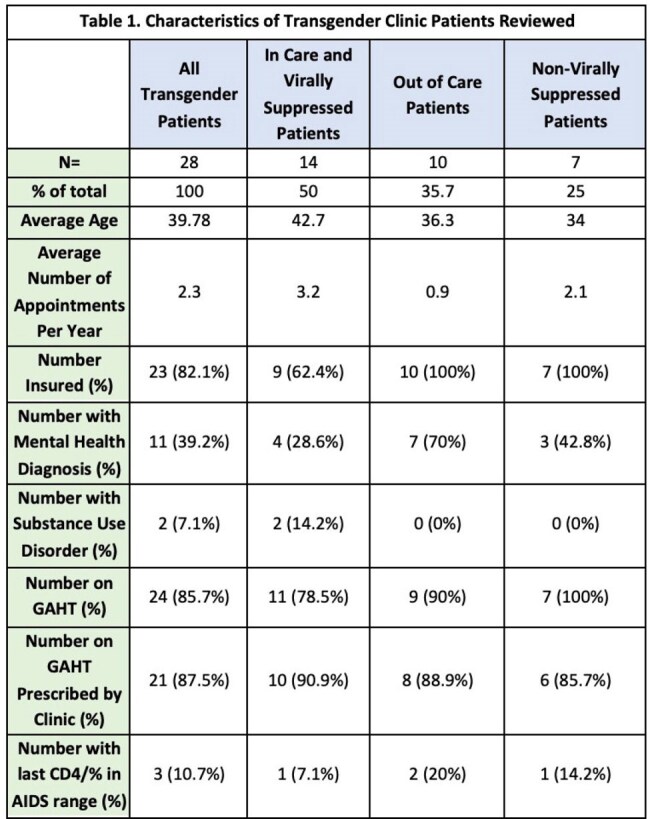
Characteristics of Transgender Clinic Patients Reviewed

**Methods:**

A list of established clinic patients with a gender identity of transgender was generated to create a database with basic demographic information, viral suppression, retention in care, and potential barriers to viral suppression or retention from April 1, 2023-March 31, 2024. Insurance status, mental health or substance use disorder, and prescription of gender affirming hormone therapy (GAHT) were documented for each patient. Patients were further subdivided into three categories “In Care and Virally Suppressed,” “Out of Care,” and “Not Virally Suppressed” for further analysis.

**Results:**

Out of 1393 established clinic patients, 28 patients were identified as transgender (2%). 27/28 of these patients are black/African American, 1 is Hispanic/Latino. 64% were retained in care, compared to 81% of all clinic patients. 75% were virally suppressed, compared to 84% of all clinic patients. Detailed characteristics are displayed in Table 1. Patients who were out of care or not virally suppressed were younger and had higher rates of mental health diagnoses. 70% of patients who were out of care carried a mental health diagnosis. There was no correlation between GAHT, substance use, or insurance status and retention in care or viral suppression.

**Conclusion:**

As suggested by previous studies, a significantly lower percentage of TWH were retained in care and virally suppressed when compared to the total clinic population, thus highlighting the need to focus on this disproportionately affected group. This TWH database will be used to track retention in care and viral suppression, and data regarding potential barriers will be used to guide quality improvement efforts among TWH, with a plan to focus on mental health services.

**Disclosures:**

**All Authors**: No reported disclosures

